# Cholesterol esterification and p53-mediated tumor suppression

**DOI:** 10.37349/etat.2023.00185

**Published:** 2023-10-31

**Authors:** Youjun Li, Michael Karin, Edward V. Prochownik

**Affiliations:** University of Kansas Medical Center, USA; ^1^Hubei Key Laboratory of Cell Homeostasis, College of Life Sciences, TaiKang Center for Life and Medical Sciences, Frontier Science Center for Immunology and Metabolism, Wuhan University, Wuhan 430072, Hubei, China; ^2^Medical Research Institute, Zhongnan Hospital of Wuhan University, Wuhan 430071, Hubei, China; ^3^Department of Pharmacology, School of Medicine, University of California, San Diego, CA 92093, USA; ^4^Division of Hematology/Oncology, Children’s Hospital of Pittsburgh of UPMC, The Department of Microbiology and Molecular Genetics, The Pittsburgh Liver Research Center and The Hillman Cancer Center of UPMC, The University of Pittsburgh Medical Center, Pittsburgh, PA 15224, USA

**Keywords:** Hepatocellular carcinoma, p53 tumor suppressor, mevalonate pathway, cholesterol esterification, sterol O-acyltransferase 1

## Abstract

Many human cancers carry missense mutations in or deletions of the tumor protein 53 (TP53) tumor suppressor gene. TP53’s product, p53 regulates many biological processes, including cell metabolism. Cholesterol is a key lipid needed for the maintenance of membrane function and tissue homeostasis while also serving as a precursor for steroid hormone and bile acid synthesis. An over-abundance of cholesterol can lead to its esterification and storage as cholesterol esters. The recent study has shown that the loss of p53 leads to excessive cholesterol ester biosynthesis, which promotes hepatocellular carcinoma in mice. Blocking cholesterol esterification improves treatment outcomes, particularly for liver cancers with p53 deletions/mutations that originate in a background of non-alcoholic fatty liver disease.

The development of hepatocellular carcinoma (HCC), the most common primary liver cancer and third leading cause of cancer-related deaths worldwide is often accompanied by the reprogramming of cholesterol metabolism. However, the precise role that this metabolic restructuring plays in HCC initiation, maintenance, and survival is poorly understood [1, 2].

Cholesterol is a key lipid needed for the maintenance of membrane function and tissue homeostasis while also serving as a precursor for steroid hormone and bile acid synthesis [3]. Excessive accumulation of cholesterol and cholesterol esters often accompanies HCC development, suggesting that this imparts a selective growth advantage and that strategies to reduce these metabolites in hepatocytes could impede HCC progression [4, 5]. Several proposed mechanisms for the pathogenic role of cholesterol in HCC have been proposed, including (1) the induction of ectopic fatty acid accumulation; (2) the re-modeling of the hepatic immune response and the establishment of a tumorigenic micro-environment; (3) the activation of hepatic stellate cells; (4) effects on membrane fluidity and protein function [6–8].

The tumor suppressor p53 [tumor protein 53 (TP53)] can inhibit the mevalonate (MVA) pathway, which is responsible for the biosynthesis of cholesterol and nonsterol isoprenoids. p53 loss or mutation plays an important role in the development of HCC by lifting numerous restrictions on tumor growth [2, 9–11]. Under low-sterol conditions, p53 transcriptionally induces the ATP binding cassette transporter A1 (ABCA1) cholesterol transporter gene, which in turn inhibits the maturation of sterol regulatory element binding protein 2 (SREBP-2), the master transcriptional regulator of this pathway. Down-regulation of MVA pathway gene expression by p53 occurs in pre-malignant hepatocytes and actively suppresses tumorigenesis in a mouse model of HCC [2]. Similarly, pharmacological or RNA interference (RNAi)-mediated inhibition of the MVA pathway restricts murine HCC development driven by p53 loss [2]. Like p53 loss, ABCA1 ablation promotes murine liver tumorigenesis and is associated with increased SREBP-2 maturation. Collectively, these findings demonstrate that MVA pathway repression is a crucial component of p53-mediated liver tumor suppression and provides mechanistic insight at the metabolic level as to why and how *TP53* gene compromise might benefit HCC progression [2].

Under normal-sterol conditions, p53 transcriptionally represses, in a SREBP-2-independent manner, squalene epoxidase (SQLE), a key enzyme in cholesterol synthesis [9]. This inhibits cholesterol production *in vivo* and *in vitro,* leading to tumor growth suppression. Inhibition of SQLE using RNAi or terbinafine (an SQLE inhibitor) reverses the cell proliferation caused by p53 compromise. Conversely, SQLE overexpression or exogenous cholesterol promotes cell proliferation. More importantly, silencing of SQLE restricts nonalcoholic fatty liver disease (NAFLD)-induced liver tumorigenesis in p53 knockout mice. Gain-of-function p53 mutants, in association with SREBP-2, bind the promoter regions of genes encoding enzymes involved in cholesterol biosynthesis, thus acting as transcriptional co-activators [12]. These findings reveal a role for p53 in regulating SQLE and other relevant genes to control cholesterol biosynthesis, at distinct points along this critical metabolic pathway and further demonstrate that its down-regulation is critical for p53-mediated tumor suppression [9–12].

In a recent paper published in *Hepatology*, a novel pathway was described by which loss of p53 alters cholesterol ester biosynthesis to promote hepatocarcinogenesis in mice maintained on a high-cholesterol and high-fat diet (HCHFD), thereby identifying a potential therapeutic target [13]. It was found that this diet potentiated hepatocarcinogenesis in a manner that was further accelerated by p53 loss. Quantitative lipidomic analysis showed that cholesterol esters were significantly elevated in p53-deficient hepatocytes. Interestingly, the re-expression of wild-type p53 suppressed tumor growth in HCHFD-fed mice and normalized the cholesterol ester imbalance. These findings suggest that one of the downstream pathways through which p53 suppresses or otherwise regulates HCHFD-induced hepatocarcinogenesis involves cholesterol ester biosynthesis.

To gain further insight into the role of cholesterol ester accumulation in hepatocarcinogenesis, several cholesterol biosynthetic enzymes were targeted and it was found that the sterol O-acyltransferase 1 (SOAT1) inhibitor avasimibe inhibited HCHFD-induced HCC induction and cholesterol ester synthesis in a p53-independent manner. Further, *in vivo* experiments showed that SOAT1 was regulated post-translationally via ubiquitin-dependent proteasomal degradation. Deubiquitinases (DUBs) play important roles in tumorigenesis by regulating ubiquitin-dependent proteasomal degradation [13, 14]. Database searches pointed to ubiquitin-specific peptidase 19 (USP19) as the likely SOAT1-specific DUB and this was confirmed by demonstrating a direct interaction between SOAT1 and USP19 in co-immunoprecipitation experiments. Furthermore, USP19 inhibition significantly reduced SOAT1 activity by increasing its ubiquitylation at relevant lysine residues. Further studies showed that p53 directly inhibited the expression of USP19 and SOAT1 at the transcriptional level.

To investigate the role of SOAT1 in hepatocarcinogenesis more directly, the generated Soat1^ΔHep^p53^ΔHep^ mice were generated and it was shown that SOAT1 was needed to support HCC growth in p53^Δhep^ mice [13]. HCCs generated in USP19 knockout mice had reduced SOAT1 that correlated with lower amounts of total cholesterol, cholesterol esters, and fatty acids. Conversely, USP19 over-expression increased SOAT1 and accelerated HCC progression.

To further test the hypothesis that USP19 loss inhibits HCC progression in response to p53 loss and that this is related to decreased SOAT1 expression, USP19 and SOAT1 expression was examined in human HCCs in The Cancer Genome Atlas database and confirmed that both messenger RNAs (mRNAs) were up-regulated. Analysis of the Human Protein Map database showed that HCC patients whose tumors contained low levels of USP19 and SOAT1 had significantly prolonged survival. Quantitative immunoblotting also showed that p53 expression negatively correlated with cholesterol ester levels in human HCCs whereas USP19 and SOAT1 proteins were up-regulated in HCC and positively correlated with cholesterol ester levels. Collectively, these findings tied together experimental findings in mice and human clinical data and lent support to the notion that the p53-USP19-SOAT1 axis is dysregulated in HCC, contributes to disease progression, impacts long-term survival, and could be a relevant therapeutic target (Figure 1). Indeed, it has recently been reported that SOAT1 is a specific marker of HCC, alters the distribution of cholesterol and promotes proliferation [5, 15]. SOAT1 inhibitors, such as avasimibe could represent a novel class of therapeutic agents, particularly for liver cancers with p53 deletions/mutations that originate in a NAFLD background [5, 13]. Similar treatments may also be applicable to other cancers [16–29].

**Figure 1:**
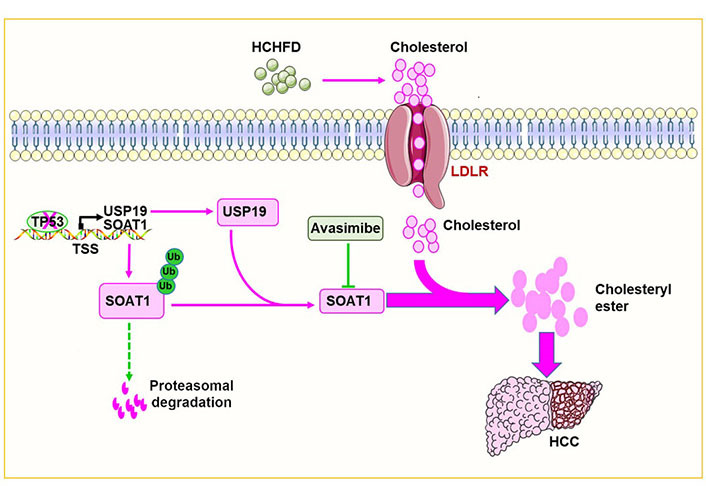
p53 deletion/mutation promotes HCC growth by increasing cholesterol esterification. p53 deletion/mutation increases the expression of USP19 and SOAT1 in hepatocytes. This in turn increases cholesterol esterification and promotes hepatocarcinogenesis. The red arrow in the figure means to promote carcinogenesis while the dotted green lines mean to inhibit carcinogenesis. ‘×’ on the TP53 indicates a deletion or mutation in the TP53. T-bar (—|): suppresses; LDLR: low-density lipoprotein receptor; TSS: transcriptional start sites; Ub: ubiquitin

SOAT1 contributes to hepatocarcinogenesis by promoting cholesterol esterification but precisely how? Cholesterol ester increases HCC tumorigenesis by promoting the synthesis of phospholipids and hormones that provide the raw materials needed for cell membrane synthesis and signaling molecules [1]. Meanwhile, SOAT1 promotes cholesterol esterification and decreases free cholesterol levels [20, 27]. Low free cholesterol could activate SREBP-1 and SREBP-2 [1, 2, 27]. SREBP-1 is responsible for lipid synthesis contributing to HCC growth [1, 2, 27] whereas SREBP-2 promotes cholesterol synthesis by regulating the MVA pathway [2, 3]. Low free cholesterol also represses liver X receptor (LXR) activity by reducing oxysterol levels that contribute to HCC [30]. Which metabolites and downstream pathways are dysregulated by SOAT1 and cholesterol ester to mediate HCC tumorigenesis is likely to be contextually dependent and will be worth investigating.

USP19 plays an important function in HCHFD-induced hepatocarcinogenesis by mediating SOAT1 protein deubiquitylation and stability [13]. This suggests that USP19 is a promising drug target and that USP19-specific inhibitors could potentially be of therapeutic utility. Also, worth investigating is whether USP19 inhibitors are promising drugs against tumors in general, or only some specific types of tumors, like liver cancers with p53 deletions/mutations that originate in a NAFLD background.

In conclusion, this study shows that p53 deletion/mutation increases USP19, which in turn stabilizes SOAT1, increases cholesterol esterification and accelerates tumor progression (Figure 1).

## Abbreviations

HCC: hepatocellular carcinoma
HCHFD: high-cholesterol and high-fat diet
MVA: mevalonate
NAFLD: nonalcoholic fatty liver disease
SOAT1: sterol O-acyltransferase 1
SQLE: squalene epoxidase
SREBP-2: sterol regulatory element binding protein 2
TP53: tumor protein 53

## Declarations

### Copyright

© The Author(s) 2023.
